# Pharmacists’ interventions improve health-related quality of life of rural older person on warfarin: a randomized controlled trial

**DOI:** 10.1038/s41598-021-01394-0

**Published:** 2021-11-09

**Authors:** Slaven Falamić, Marko Lucijanić, Maja Ortner-Hadžiabdić, Srećko Marušić, Vesna Bačić-Vrca

**Affiliations:** 1 Pharmacy Branka Marušić, Trg Ante Starčevića 24, 31450 Donji Miholjac, Croatia; 2 Hematology Department, University Hospital Dubrava, Avenija Gojka Suska 6, 10000 Zagreb, Croatia; 3 Centre for Applied Pharmacy, Faculty of Pharmacy and Biochemistry, University of Zagreb, Ante Kovačića 1, 10000 Zagreb, Croatia; 4 Department of Clinical Pharmacology, University Hospital Dubrava, Avenija Gojka Suska 6, 10000 Zagreb, Croatia; 5 Department of Clinical Pharmacy, University Hospital Dubrava, Avenija Gojka Suska 6, 10000 Zagreb, Croatia

**Keywords:** Health care, Health occupations

## Abstract

Warfarin therapy can significantly affect patients’ quality of life and cause therapy discontinuation. This study aimed to investigate the effect of the pharmacists’ interventions on the health-related quality of life (HRQoL) in older rural patients on warfarin therapy. Eligible older patients from rural area of Croatian province Slavonia were randomized into the intervention and control groups and followed for six months. Repeated education and a follow-up plan were provided to the participants in the intervention group, and if needed, the pharmacist intervened to optimize warfarin therapy. Secondary analysis on HRQoL data are presented here. Main outcome measure was Duke anticoagulation satisfactions scale questionnaire score. In total, 131 participants finished the study (median age 73 years; 51.1% male). Participants in the intervention group scored significantly lower (median being 86.5 and 66.0 in the control and intervention groups, respectively; p < 0,001), indicating higher HRQoL. Adverse drug reactions and pharmacist’s intervention were identified as predictive factors for patients’ HRQoL (r^2^ = 65.5%, P < 0.001). The study demonstrated that community pharmacist’s interventions can improve HRQoL of older patients taking warfarin what is of particular significance for patients living in rural areas with less accessible healthcare and lower socio-economic status.

Clinicaltrials.gov (ID: NCT03212898), 11/07/2017, retrospectively registered.

## Introduction

Warfarin is a widely prescribed anticoagulant drug for indications such as atrial fibrillation, cardiac valve replacement, deep vein thrombosis, and pulmonary embolism; although it has been gradually replaced by direct oral anticoagulant drugs (DOACs)^1^.

Warfarin therapy could be demanding for the individuals, who are mostly older patients. Frequent visits to the health facility for blood sampling to measure INR, dietary recommendations to avoid warfarin-food interactions, and safety issues due to drug-drug and drug-disease interactions make it difficult for patients to adhere to warfarin therapy. Moreover, older patients are more sensitive to warfarin and had a higher risk of major haemorrhagic events as compared to younger patients. Reasons for that are both age related changes contributing to pharmacokinetic changes as well as increase in comorbid conditions and concomitant medication use in older people^2^. Nonetheless, long warfarin use, a well-established efficacy and safety profile and an affordable price make it the drug of choice for many older patients. This is especially true in countries where warfarin is covered by health insurance, and patients do not participate in the costs of warfarin therapy, compared to DOACs.

Warfarin-related research has shown that individuals in the lowest socio-economic status category and those living in rural areas were at a significantly increased risk of elevated INR levels^3^. Further, older rural patients tend to have lower socio-economic status and educational level and often have limited access to healthcare^4^. Moreover, there is concern that some patients, particularly those from a rural location, may not perceive a significant benefit in transitioning from warfarin to DOACs or may have concerns and therefore are not good candidates for being prescribed DOACs^5^. All these suggest increased safety issues for older patients on warfarin therapy living in rural areas.

Health related Quality of Life (HRQoL) is influenced by numerous factors among which warfarin therapy^6^ as well as living in rural area with limited access to healthcare has been identified as negative predictors for HRQoL^7,8^. We hypothesize that community pharmacist’s interventions could have beneficial effect on HRQoL in older rural population on warfarin. Literature describing impact of different interventions on HRQoL in older rural warfarin patients is scarce and to the best of our knowledge no randomized controlled studies are available. An observational study found that the HRQoL of patients taking warfarin over one year declined significantly^9^, which is not surprising given the side effects, periodic monitoring that entails discomfort to the patient, and other therapy limitations. Reviews assessing pharmacists’ interventions on older adults to improve HRQoL provide mixed findings and suggest the need for further research on this regard^10,11^. Clarkesmith et al. suggest that it is important to explore the psychological implications for patients experiencing long-term anticoagulant therapy^12^. Thus, the aim of this study was to perform a randomized controlled trial to investigate the influence of the pharmacists’ intervention on the HRQoL in older rural patients consuming warfarin. This was a planned secondary aim and analysis, building on our previously published findings on positive pharmacist’s interventions on time in therapeutic range (TTR)^13^ and adverse reactions in patients taking warfarin^14^.

## Methods

### Study design, location of study and sample selection

The study was registered at Clinicaltrials.gov (ID: NCT03212898) and complies with the CONSORT statement. It was designed as a randomized controlled trial and conducted at a community pharmacy in a rural region of Croatia (Donji Miholjac). The patients were randomized into the intervention and control groups using a computer-generated randomization program (www.randomization.com). Patients and their GPs were blinded and did not know which group the patient belonged to. The pharmacist who was responsible for providing interventions and monitoring patients because of the nature of the intervention was unmasked.

The inclusion criteria were (1) 65 years of age or older; (2) rural place of residence; (3) taking warfarin at least three months before enrolling in the study with an expected duration of at least a further six months of prescription. The exclusion criterion was prescribed interacting drug with X degree of clinical significance based on the Lexi®-interact database. Written informed consent was obtained from every eligible patient. More details on study design and protocol are available in previously reported results of the study which encompass primary and co-secondary outcome measures related to the effectiveness and safety of the therapy ^13,14^. Herein we present secondary analysis data on HRQoL, measured by Croatian-adopted DASS (Duke anticoagulation satisfaction scale) questionnaire.

### Ethics approval

Ethical approval (number 251–62-03–15-28) was obtained from the Ethical Committee of the Faculty of Pharmacy and Biochemistry, University of Zagreb. The trial was performed in accordance with the international, national and institutional guidelines pertaining to clinical studies and biodiversity rights and it also complies with the CONSORT guidelines.

### Data collection and intervention

During the initial visit, the clinical pharmacist interviewed the patients and collected data on the patients’ sociodemographic characteristics, clinical data, and medication history. Data on comorbidities, indication for pharmacotherapy, and International Normalized Ratio (INR) were additionally collected from medical records provided by GP and data available in the pharmacy database. During monthly follow-up visits, the clinical pharmacist reviewed the medication therapy and assessed the laboratory and physical examination results from the medical records. If unable to come to the pharmacy, patients were phoned by the investigator for data collection and counselling on the most important issues. Caregivers were not educated during the study period. TTR was calculated using the Rosendaal method ^15^. The patients were asked about their experience of adverse drug reactions, consumption of food rich with vitamin K (i.e. kale, collard, spinach, parsley, endive, cabbage etc.), and their adherence was measured by the number of pills left in the pillbox or pill package. All study participant completed the DASS questionnaire at the end of the study (after six months).

Pharmacist’s intervention provided to the treatment group entails: (1) medication review, (2) identification of the drug related problems focusing on warfarin therapy, including drug interactions and non-adherence issues and (3) depending on the individual case, patient consultation and/or therapy optimisation recommendations to the GP.

The pharmacist interacted one-to-one with the patient for 45 min providing education on different aspects of warfarin therapy. Moreover, patients in the intervention group were provided with a follow-up plan and pillbox, if needed. Follow-up visits were scheduled monthly in the community pharmacy and lasted for about 20 min. Interventions for warfarin dose change and the avoidance of drug interactions were recommended and discussed with the general practitioners (GPs).

Control group received standard care which consists of regular visits to the GP and visits to the community pharmacy to obtain the prescribed medicines. GP provided routine care to patients on warfarin, which included determining the frequency of monitoring INR and subsequent actions, such as dose changes or patient counselling. There was no regular consultation or collaboration between the GPs and community pharmacist The follow-up period for the control group was six months.

### Measurement of health-related quality of life

We used the Croatian-adapted DASS questionnaire (Cro-DASS) to measure the HRQoL of the enrolled patients. DASS is a specific scale to evaluate the HRQoL of patients taking oral anticoagulants. The questionnaire has 25 items and contains three domains: (1) limitations (9 items); (2) hassles and burden (8 items) and 3) psychological impacts (8 items). Each item in the questionnaire is assessed using a 7-point Likert scale (ranging from “not at all” to “very much”). The overall score varies from 25 to 175 (9 to 63 scores for limitations domain and 8 to 56 scores for both hassles and burden domain and psychological impacts domain). The items presenting the lowest scores correlate with higher satisfaction and HRQoL. Items that were negatively worded were reverse coded during the data entering into the database (items 19, 21, 22, 23, 25, 27). Croatian version of the questionnaire was developed for the purpose of this study and the adaptation process ensured that it was conceptually equivalent to the original English version. It involved linguistic validation and cultural adaptation. None of the questions was omitted or altered. Cro-DASS questionnaire was applied using a paper form by the pharmacist.

### Statistical analysis

Normality of variable distribution was assessed using the Kolmogorov–Smirnov test. Data were presented as percentages (%) for categorical variables and median and interquartile range for non-normally distributed numerical variables. Differences in baseline characteristics between the intervention and control groups for interval variables were assessed using the Mann–Whitney U-test, and comparisons between proportions were assessed using the chi-square test. The contribution of each independent variable (TTR, number of comorbidities, age, gender, ADR appearance, adherence rate, educational level, and pharmacists’ intervention) to the overall Cro-DASS scores was evaluated using a multiple regression model. A two-sided p value of less than 0.05 was considered statistically significant. Statistical analysis was performed on IBM SPSS Statistics version 25 (IBM Corp., Armonk, NY, USA).

## Results

### Baseline patients’ characteristics

Of the 160 patients, who met the inclusion criteria, 140 agreed to participate in the study and were randomized to the intervention (n = 70) and control (n = 70) groups. Nine patients dropped out; five in the intervention group and four in the control group. Hence, 131 patients were included in the final analysis. Median age of the study participants was 73 (IQR 70–80) years; 71.8% were between 65 and 80 years, and 28.2% were 80 years and older. The study population consisted of 51.1% of male participants, most of whom had only basic level of education (elementary school, 67.2%) and were married (49.6%). The most common indication, among these participants, for warfarin use was atrial fibrillation (74.1%), while the median number of comorbidities was 4 (IQR 4–5) and median number of drugs in the therapy was 6 (IQR 5–7). The characteristics of the participants did not differ significantly between the groups and are presented in Table 1.

**Table 1:** Baseline sociodemographic and clinical characteristics of the study participants.

Variable	All participants (n = 131)	Control group (n = 66)	Intervention group (n = 65)	P
Age (years)	73 (70–80)	74.5 (71–81)	72 (68–79)	0.053
Gender (male)	67 (51.1%)	34 (51.5%)	33 (50.8%)	0.932
**Indication**				
Atrial fibrillation	97 (74,1)	47 (71.2%)	50 (76.9%)	
Phlebothrombosis	12 (9.2%)	6 (9.1%)	6 (9.2%)	
Other	22 (16.8%)	13 (19.7%)	9 (13.8%)	0.456
Number of comorbidities	4 (4–5)	4 (4–5)	5 (3–5)	0.408
Number of drugs in therapy	6 (5–7)	6 (5–7)	6 (5–8)	0.187
**Education**				
No formal education or elementary school	88 (67.2%)	47 (71.2%)	41 (63.1%)	0.260
High school	37 (28.2%)	15 (22.7%)	22 (33.8%)	
College/University	6 (4.5%)	4 (6.1%)	2 (3.1%)	

Data are presented as n (%) or median (interquartile range). P values obtained by Mann–Whitney U test for numerical data or Chi-squared test for categorical variables.

### Patients’ satisfaction and HRQoL with warfarin therapy at six months

Results of the Cro-DASS questionnaire for the intervention and control groups are presented in Table 2, depicting final as well as individual domain scores. There were statistically significant differences in all three domains, namely limitations, hassles and burden, and psychological impacts, as well as in overall scores between the randomization groups. Participants in the intervention group scored lower in all domains, indicating higher satisfaction and HRQoL related to the use of warfarin compared to that of the control group.

**Table 2:** Cro-DASS scores at the end of the study period (after six months).

DASS domain	Control*	Intervention*	P
Limitations	23.0 (21.0–26.0)	17.0 (16.0–18.8)	< 0.001
Hassles and burden	23.0 (19.0–25.0)	16.0 (15.0–18.0)	< 0.001
Psychological impacts	41.0 (35.0–43.0)	33.0 (32.0–35.0)	< 0.001
Overall Cro DASS score	86.5 (77.0–94.0)	66.0 (63.0–69.0)	< 0.001

*Median score (interquartile range) on scale 25 to 175 scores for overall Cro-DASS, 9 to 63 scores for Limitations domain, 8 to 56 scores for Hassles and burden domain and 8 to 56 scores for Psychological impacts domain; lower scores indicating a better quality of life.

P value calculated using the Mann–Whitney U test.

### Predictors of patients’ satisfaction and HRQoL with warfarin therapy

Results of the multivariate logistic regression model are presented in Table 3. Of the nine variables entered in the regression model, only ADRs and pharmacist’s intervention were identified as predictive factors (r^2^ = 65.5%, P < 0.001), among which experience of ADRs being the strongest predictor for higher Cro-DASS scores (lower satisfaction), accounting for 52.7% of variance of the overall model. Experiencing ADRs and not being exposed to the pharmacist’s intervention were positive predictors of lower satisfaction with warfarin therapy after the follow-up period.

**Table 3:** Multiple regression for Cro-DASS scores at six months.

Variable	Unstandardized (B) coefficient	Standard error	Standardized (beta) coefficient	P
TTR	0.021	0.042	0.049	0.619
Number of comorbidities	−0.108	0.709	−0.008	0.879
Age	0.253	0.161	0.108	0.119
Gender (female)	2.086	1.873	0.070	0.267
Education level (higher)	−1.408	1.733	−0.054	0.418
Adherence	−0.073	0.108	−0.051	0.500
Pharmacist's intervention	−9.513	2.878	−0.317	0.001^a^
ADR	11.435	1.587	0.527	< 0.001^a^
Marital status (non-married)	0.380	2.183	0.013	0.862

R^2^ = 65.5%; adjusted R^2^ = 63.0%, p < 0.001.

^a^Statistically significant.

## Discussion

To the best of our knowledge, this is the first randomized controlled trial, conducted to assess the effects of community pharmacists’ intervention on the HRQoL of older adult patients taking warfarin, in rural areas. The study demonstrated that a pharmacist’s intervention had a positive impact on the HRQoL in the investigated population. This is an important finding that builds on our previously published primary and secondary analysis of the study, showing that HRQoL in addition to other three explored outcomes (TTR, adherence and ADRs) differed significantly among the groups and all explored outcomes were more favorable in the intervention group (Supplementary table).

It is difficult to compare our results with those of other studies because reports of studies with similar characteristics in terms of the aim and research design are not available. Therefore, we have discussed our results by comparing them with the results of studies that included different populations or settings. A meta-analysis which included 48 studies, found that pharmaceutical care interventions can significantly improve at least one domain of HRQoL^16^. However, only one of the included studies involved patients on anticoagulation; it was performed in a tertiary care setting and compared pharmacist-led warfarin self-management program with standard care at the anticoagulation clinic and found the benefits of the program^17^. A study exploring cardiologist interventions has found that the efficiency of warfarin increased significantly, but surprisingly, HRQoL and the patients’ satisfaction with warfarin use deteriorated significantly after regular education and follow-up in patients from rural areas^18^. This is contrary to our results showing that both the efficiency, measured by TTR^13^ and HRQoL increased in intervention group. This could be explained by the differences of the study population but also the fact that pharmacists provide pharmaceutical care which differs from medical care provided by cardiology specialist might have contributed to the observed results. The concept of pharmaceutical care implies that pharmacist makes a direct, personal, caring commitment to the individual patient and acts in the patient’s best interest. The pharmacist cooperates directly with other professionals and the patient in designing, implementing, and monitoring a therapeutic plan intended to produce definite therapeutic outcomes that improve the patient’s quality of life^19^. We believe that it is the concept of pharmaceutical care and its core element patient-centred approach that has contributed to the positive results of our study. It supports the involvement of patients in an informed way, in establishing quality-of-life goals for their therapies. That a patient-centred approach leads to a better quality of life was also confirmed by a systematic review by Rathert and co-authors. They reported that more patient-centred organisations also have more positive outcomes, one of them being greater quality of life and well-being of patients^20^.

Several studies have addressed the topic of frequency of visiting healthcare providers and facilities and their influence on the quality of life. Matchar et al. found that the HRQoL increases when the patient is less dependent on a visit to the doctor and the laboratory and can monitor the INR at home using a portable device (POCT, point-of-care testing)^21^. Authors also emphasized that such testing does not affect clinical outcomes or mortality. Similarly, Sølvik et al.^22^ reported an improvement in the HRQoL of patients who monitored INR and titrated warfarin doses by themselves. According to the authors, patients who switch from conventional care to the self-care model after two years have a significantly better HRQoL, probably because they are less dependent on visiting health facilities. Contrarily, Carris et al. found that anticoagulation satisfaction following extended interval monitoring, as measured by the DASS survey, did not change, and may have marginally worsened following extended interval follow-up^23^. They suggest that less frequent feedback and patient–provider interaction may have resulted in reduced patient perception of benefit from anticoagulation and reduced self-management activities^23^. We agree with the last observation and believe that the intervention group in our study benefited from more frequent patient-provider interaction. We believe that the time and attention invested in the intervention group were one of the key factors influencing the results.

Our secondary aim was to identify factors predictive of HRQoL. We found that among the nine analyzed variables only pharmacist intervention and experience of the ADRs were predictive factors. Experience of the ADRs was identified as the strongest predictor for lower HRQoL. Hemorrhagic events negatively influenced HRQoL in Lancaster et al.’s study^24^ but did not have an influence on HRQoL in Casais et al.’s study^25^. Although other studies reported different findings on the association between ADRs and HRQoL, based on our results, we recommend focusing on the management of ADRs to increase HRQoL in patients on warfarin. As most of the recorded ADRs in our sample were not serious (i.e., bruising), we believe that counselling patients on minor bleeding signs would increase patients’ satisfaction with warfarin therapy. Other studies identified socio-demographic factors to influence HRQoL in patients on warfarin^9,26^. Although Hasan et al. have shown that men score higher^9^, this effect has not been confirmed in our study. Neither the years of consumption of warfarin affected the HRQOL in our study, unlike other studies^9,26^, probably because our sample was more homogeneous by including only older adults. Almeida et al. have published the result of average DASS score in a cohort of 67 Brazilian patients and found that the HRQoL was influenced by experience of side effects, age, comorbidities, drug interactions, level of education, and length of treatment^26^.

We believe that our results significantly contribute to the HRQoL and pharmaceutical care research, as this is the first study using randomized controlled design exploring the effect of community pharmacists’ intervention on HRQoL of older rural patients on warfarin. Measuring humanistic outcomes in the form of HRQoL, in addition to clinical and economic outcomes, provide a comprehensive picture of the impact of medicine or healthcare interventions^27^. Improving or maintaining patients’ HRQoL is a fundamental goal of the pharmaceutical care services^16^.

Depending on the country’s development, there may be barriers in providing quality healthcare and clinical pharmacist services to rural area, as identified by Patterson et al. in a sample of more than 3 million patients^28^. Patients consuming warfarin require frequent visits to the laboratory and doctor, which can be difficult in rural areas. Even in well-developed countries and health care systems, such as in the USA, urban patients with atrial fibrillation received warfarin more often than those in rural areas, although the risks of stroke in both groups were similar^29^. A large study conducted by Rose et al. on 56,490 older patients showed that there was a loss of follow-up for such patients and that they did not perform regular laboratory monitoring, and the risk of loss of follow-up was associated with race, poverty, dementia, depression, and remoteness^30^. The present study highlights that community pharmacists are important elements of the health care system, who can help in overcoming the barriers in providing care to patients on warfarin.

These observations have important clinical implications for rural regions and are particularly applicable to the countries where pharmacists-managed anticoagulation monitoring services are not implemented. Patients living in socially deprived regions, who need support in their warfarin therapy, and do not have the access to health care facilities could benefit from community pharmacist managed service. We strongly believe that a well-educated, competent, sufficient and well distributed pharmacy workforce should be a priority for wider applicability of intervention explored in our study, as well as the recognition and reimbursement of pharmacy services. This is particularly important for vulnerable older patients taking high risk drugs and in areas with low access to other healthcare facilities than community pharmacy.

The study has several limitations. Firstly, since the HRQoL instrument was not applied before and after the pharmacists’ intervention, we could not observe the magnitude or the direction of the change of the HRQoL, although randomized controlled design of our study allowed observing positive effect in the intervention group. This study presents the results of a secondary outcome data; therefore, power analysis was not performed for the HRQoL outcome, but it was performed for previously published primary outcome of the study^13^. Moreover, in interpreting the results, one should be aware of the extent of care provided to patients in the intervention group compared to the control and the fact that patients potentially may have been unblinded during the process could have caused bias.

Furthermore, it is important to emphasize that the patients allocated to the intervention received a multicomponent, multilevel intervention. The intervention consisted of medication review, counselling and patient education, providing patients with a follow-up plan and a pill box and therapy modification in collaboration with the GP. There is no manner to determine which of the actions performed had the most significant impact on the results. We argue that it is a comprehensive, complex intervention, whose important features are time and attention devoted to the patient, which is responsible for the results obtained.

Our results are applicable to a specific population- elderly rural patient. The isolation of these patients, low level of education, and low availability of health care could have created a stronger positive impact of pharmacy interventions in the study patients.

Based on our findings, we suggest that a well-educated, competent, sufficient and well distributed pharmacy workforce should be a priority for wider applicability of the intervention explored, but also the recognition and reimbursement of pharmacy services. This could be of particular value for vulnerable older patients taking high risk drugs and in areas with low access to other healthcare facilities than community pharmacy.

This study demonstrated that pharmacist’s interventions conducted at a community pharmacy can positively affect the HRQoL of older adult patients on warfarin therapy. As the evidence relates to the rural region, these results indicate that community pharmacists as the most assessable health-care professionals could play an important role for rural patients. Moreover, the results of this study indicate the importance of focusing on ADRs related to warfarin therapy to positively affect the patient’s HRQoL.

## Supplementary Information

Supplementary Information.
