# Supplementary material for: Pharmacists’ interventions improve health-related quality of life of rural older person on warfarin: a randomized controlled trial
Source: Sci Rep. 2021 Nov 9;11:21897. doi: 10.1038/s41598-021-01394-0 (PMC8578616; doi:10.1038/s41598-021-01394-0)
Supplement: Supplementary file 1 — Supplementary Information. [file 41598_2021_1394_MOESM1_ESM.docx]

Supplementary table: ADR incidence, patients’ adherence to the warfarin therapy, and estimated TTR at the end of the follow-up period [13,14]

| **Variable** | **Control group** | **Intervention group** | **p** |
| --- | --- | --- | --- |
| **TTR** | 31.2 (0-50.2) | 93 (71.7-100) | <0.001^a^ |
| **ADRs n (%)** | 56 (85%) | 19 (29%) | <0.001^b^ |
| **Adherence** | 69.0 (64.3-72.5) | 80.2 (75.9-87) | <0.001^a^ |

^a^ P value calculated using the Mann-Whitney U test

^b^ P values calculated using the chi-square test for categorical data

Data summary from published work by Falamić et al. [13,14]
